# Academic–Industrial Collaboration in Synthesis with Real-Time Impact in Medicinal Chemistry: Discovery of Cystic Fibrosis C2 Corrector ABBV-602

**DOI:** 10.1021/acsmedchemlett.5c00456

**Published:** 2025-10-13

**Authors:** Eric A. Voight, Wei Gong, David J. Hardee, Timothy R. Hodges, Michael R. Schrimpf, Stephen P. Lathrop, Jack C. Sharland, Bo Wei, Huw M. L. Davies

**Affiliations:** † Discovery Research, 359181 AbbVie Inc., 1 North Waukegan Rd., North Chicago, Illinois 60064, United States; ‡ Process Chemistry, AbbVie Inc., 1 North Waukegan Rd., North Chicago, Illinois 60064, United States; § Department of Chemistry, 1371 Emory University, 1515 Dickey Drive, Atlanta, Georgia 30322, United States

**Keywords:** enantioselective cyclopropanation, cystic fibrosis, dirhodium tetracarboxylate, donor/acceptor carbene, drug discovery

## Abstract

This article highlights synergistic real-time interactions between academic and industrial groups that drove innovations in both medicinal chemistry and catalysis. An AbbVie medicinal chemistry team had identified a promising series of trisubstituted cyclopropanes during a drug discovery campaign focused on developing CFTR C2 correctors for the treatment of cystic fibrosis. However, this unique chemical space was challenging to efficiently explore due to known limitations with previously established cyclopropanation reaction conditions. By expanding upon an existing precompetitive relationship with the Davies group at Emory University who are pioneers in development of methods for highly diastereo- and enantioselective cyclopropanations, a joint industry-academia team collaborated to discover a unique catalyst-additive system for this challenging transformation that had a broad and pharmaceutically relevant substrate scope. The optimized method was immediately applied to accelerate medicinal chemistry progress in the series, leading to the identification of novel CFTR corrector ABBV-602. Finally, a flow procedure was developed for generating the carbene precursor which enabled the reaction to be carried out on kilogram scale.

## Graphical abstract

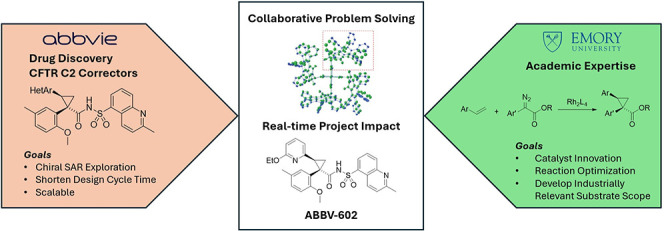

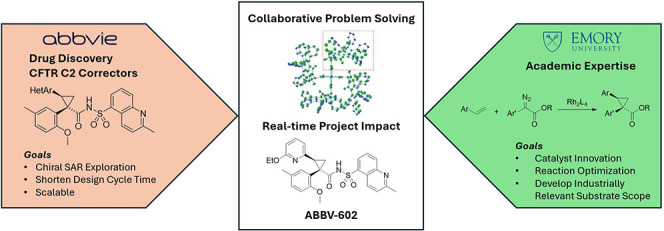

Over the years, advances in transition metal catalyzed reactions have had a tremendous impact on the enabling synthetic methodologies used to generate drug candidates in the pharmaceutical industry. Once the potential of a powerful new strategically important method has been recognized, extensive efforts have been made to maximize its utility, as seen most notably with Nobel prize winning reactions: metal-catalyzed cross coupling,^1^ asymmetric hydrogenation^2,3^ and metathesis.^4^ In order to maximize the chances of bringing new strategic reactions to bear on pharmaceutically relevant synthetic challenges it is advantageous to encourage communication between academic and industrial researchers. This paper highlights the role of a collaborative research community consisting of multiple academic and industrial groups that led initially to collaborative precompetitive research and then eventually to a critical transformation required for the generation of advanced drug candidates for the treatment of cystic fibrosis.

In 2012, the NSF funded the Center for Selective C–H functionalization (NSF-CCHF), consisting of 25 research groups from 15 universities.^5^ Its mission was “to leverage its collaborative potential to develop technology for selective C–H functionalization that will revolutionize the practice and reshape the teaching of chemical synthesis, empowering end users in materials science, fine chemicals development, and drug discovery.” In addition to the 25 academic groups, several companies became members of the CCHF, which encouraged discussions on how to translate C–H functionalization into pharmaceutically relevant applications. The collaborative environment was highly productive, generating over 300 publications with the majority being collaborative with multiple senior investigators as coauthors. After 10 years, the CCHF reached its programmed termination, but there was great interest in continuing a collaborative and synergistic research community. Consequently, in 2021, a new community, called the Catalysis Innovation Consortium (CIC), covering catalysis more broadly, was started with the general aspirations illustrated in Figure 1. Academic interest in being part of this consortium has been vast, and currently we have 44 research groups from 28 universities involved as well as several industrial partners.

**Figure 1:**
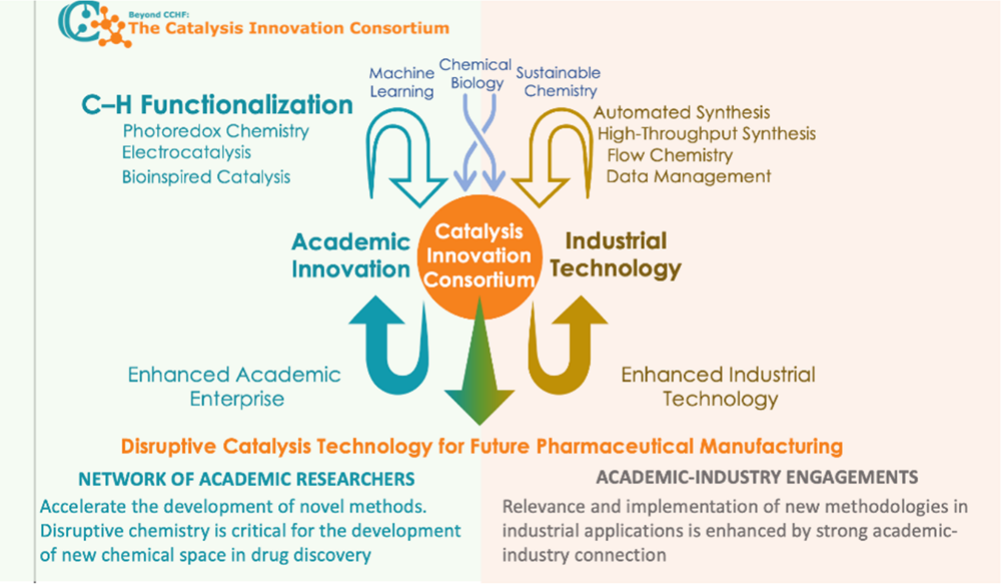
Illustration of the goals of the Catalysis Innovation Consortium. Enhancing the chemical research infrastructure by combining the chemical innovations in academia with the technological expertise and chemical challenges in industry.

In order to bring to fruition the translational potential of the research developed in the CCHF and CIC, it was necessary for both the academic and industrial partners to think beyond their normal research boundaries and find new areas for collaboration. AbbVie, under the leadership of Eric Voight, became an early partner of the CCHF and began looking for C–H functionalization projects that could be of interest to them. Huw Davies, as the Director of both the CCHF and the CIC, was also open to collaboration with both academic and industrial partners. His program focuses on the rhodium-catalyzed reactions of donor/acceptor carbenes.^6^ Even though these carbene intermediates are capable of a range of highly diastereoselective and enantioselective transformations, for them to be useful in an industrial setting two major challenges would need to be overcome: the high energy of the diazo compounds (the precursors to the carbenes), and the expense of the dirhodium catalysts. Encouraged by the industrial partners in the CCHF and CIC, the Davies group has been involved in collaborative projects to mitigate these challenges by generating the diazo compounds in flow^7–9^ and using extremely low catalyst loading.^10,11^

In the drug discovery space, AbbVie became interested in determining what types of novel chiral scaffolds could be generated by the Davies C–H functionalization approach. The two groups began a precompetitive collaboration in which the studies were broadly discussed in the Center as the work progressed. During these studies, a palladium catalyzed C–H functionalization method was developed to generate a broad collection of aryldiazoacetate carbene precursors (Figure 2A).^12^ These aryldiazoacetates were then demonstrated to be capable of highly stereoselective synthesis of silacyclobutane and silacyclopentane derivatives (Figure 2B).^13^ Then, the project was expanded to the C–H functionalization of arylcyclobutanes, in which site selectivity could be controlled by using suitable catalysts; an uncrowded catalyst favors the tertiary benzylic site whereas a bulky catalyst favors C–H functionalization at the C3 secondary site (Figure 2C).^14^ During these studies, the AbbVie group became familiar with the chemistry of the donor/acceptor carbenes and realized the practical utility of this chemistry. The catalysts are air and moisture stable, very active at carbene generation from diazo compounds, and the reactions do not require specialized equipment. Even though the aryldiazoacetates are high energy compounds, they are relatively easy to handle, although great caution would be required for any large-scale synthesis.^15^

**Figure 2:**
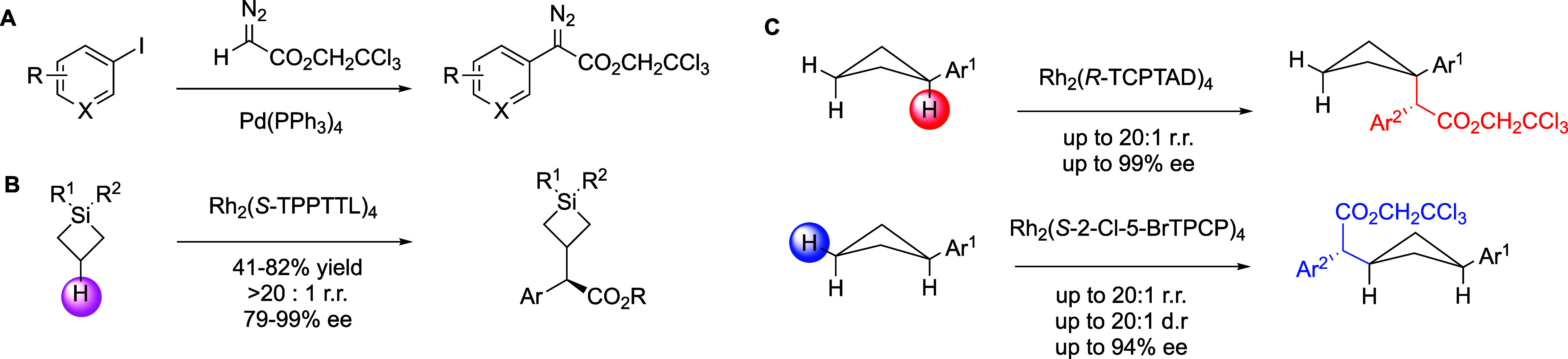
Summary of the early precompetitive collaborations between AbbVie and the Davies groups. A. Synthesis of aryldiazoacetates by palladium catalyzed cross coupling. B. C–H functionalization of silacyclobutanes. C. Catalyst-controlled site selective C–H functionalization of arylcyclobutanes.

While these collaborative projects were ongoing, AbbVie was engaged in an internal drug discovery program to develop correctors for the treatment of cystic fibrosis (CF). CF is a life-altering genetic disease resulting from mutations in the gene for the cystic fibrosis transmembrane conductance regulator (CFTR), an anion channel widely expressed in epithelium that regulates electrolyte transport and hydration of airways.^16^ Genetic abnormalities in *CFTR* produce proteins that are misfolded, unstable or poorly functional, leading to the serious respiratory and digestive symptoms that are hallmarks of the disease. The most prevalent of these mutations is deletion of F508 which affects 85% of CF patients.^17^ Over the past decade, tremendous progress has been made in the treatment of CF, including the F508del mutation, by using combinations of small-molecule modulators that alter the expression and function of CFTR channels.^18^ Typically, the drug combination includes a “potentiator” component that increases CFTR channel opening and two mechanistically distinct “correctors” (arbitrarily called C1 and C2) that improve protein stability and trafficking.

As part of a program to develop a triple-combination drug therapy for treatment of CF, we explored a series of acylsulfonamide C2-correctors containing an aryl cyclopropane carboxylate motif, exemplified by **1** (Figure 3A). Newly synthesized corrector molecules were evaluated for their ability to increase CFTR protein expression at the cell surface^19^ and enhance ion transport through native CFTR channels in human bronchial epithelial cells obtained from CF patients.^20^ During this work we found that addition of a second phenyl group to the cyclopropane (**2**) unexpectedly improved corrector potency.^21^ Resolution of racemic **2** demonstrated that (*S,R*) antipode **3** is more potent in the HBE-TECC assay and induces higher levels of CFTR cell surface expression than (*R,S*) **4**. This result prompted us to further investigate the SAR around this novel substitution pattern.

**Figure 3:**
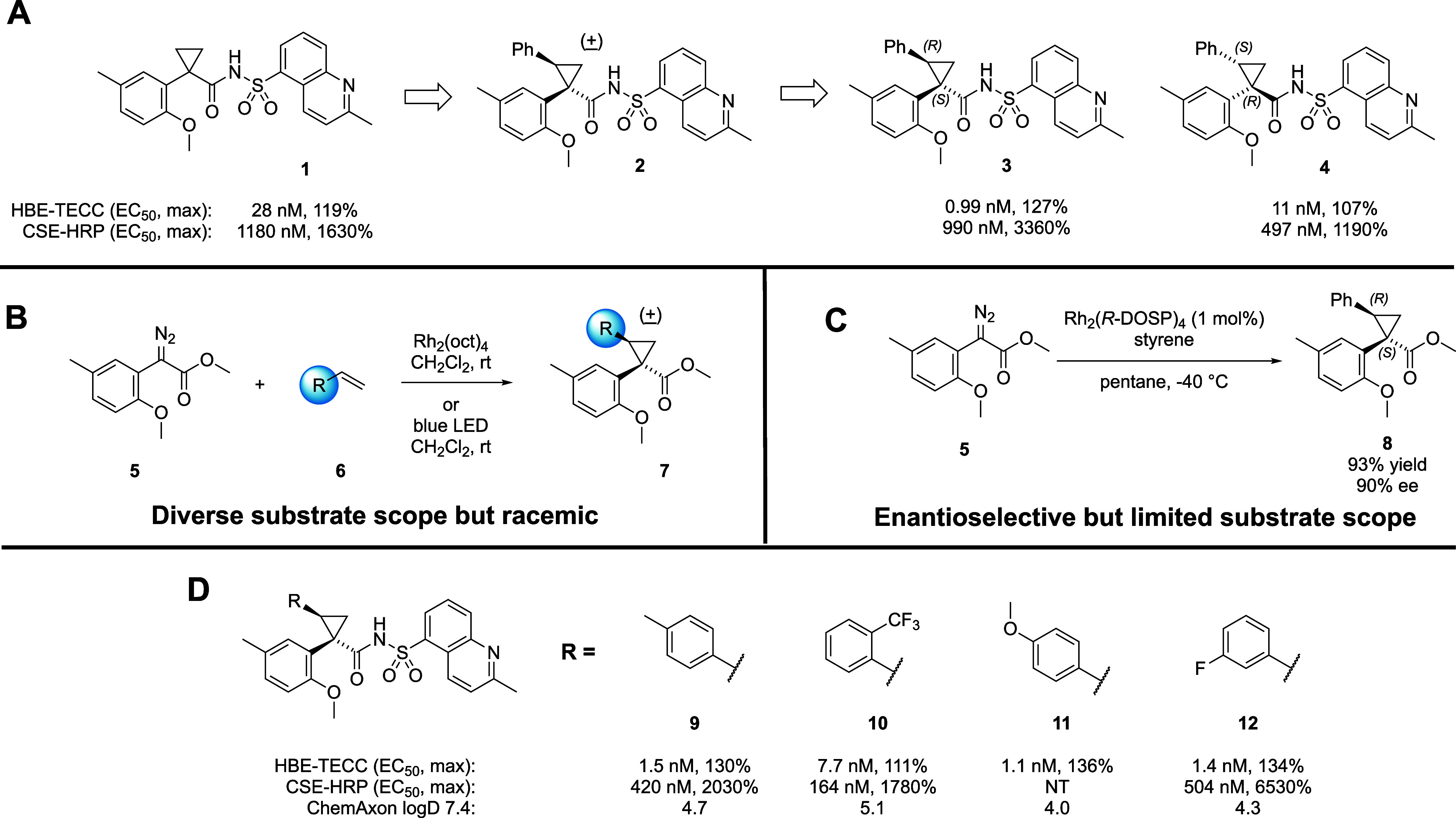
Summary of early SAR and synthetic approaches. A. Appending a phenyl group to the core cyclopropane moiety of **1** improved CFTR C2-corrector activity leading to diaryl cyclopropanecarboxylates as lead structures with preference for the stereochemical configuration of **3**. B. Early SAR studies were conducted in a diastereoselective but racemic fashion, relying on chiral SFC separation as a final step to afford enantioenriched analogs. C. Enantioselective Rh-catalyzed cyclopropanation afforded key intermediate **8** in high yield and %ee, but the reactivity proved too substrate specific for efficient enantioselective SAR exploration. D. Early substituted cyclopropane SAR generated via racemic synthesis and subsequent chiral SFC separation.

A particularly attractive feature of these new lead compounds is that the cis orientation of the two aryl rings is readily accessed using the Davies cyclopropanation with donor/acceptor carbenes. The rhodium-catalyzed reaction is highly diastereoselective, routinely favoring the cis diarylcyclopropane in >20:1 dr.^22,23^ Furthermore, Davies has shown that high cis diastereoselectivity can be achieved if the reaction is carried out under thermal conditions without catalysts^24^ or under blue-light initiated conditions.^25^ This approach provided high control of relative stereochemistry on the cyclopropane and enabled the synthesis of early proof-of-concept designs. However, during the early stages of this project, compounds such as **3** and **9**–**12** were synthesized by a racemic route followed by resolution using chiral SFC chromatography (Figure 3B and 3D). Because the SFC chromatography resource is shared among project teams at AbbVie, we were required to add our new analogs to a queue, leading to delays and limiting throughput.

Fortunately, Davies’ first-generation catalyst Rh_2_(*R*- or *S*-DOSP)_4_ ^26^ has been found to be very effective for the asymmetric cyclopropanation of styrene with methyl aryldiazoacetates and generated the desired absolute configuration of these new C2-corrector analogs.^22,23^ We quickly found that Davies’ Rh_2_(*R*-DOSP)_4_ catalyst system provided the desired cis cyclopropane **8** precursor to **3** in high yield (93%) and with excellent stereoselectivity (>40:1 dr, 90% ee), as shown in Figure 3C. This chemistry enabled the synthesis of larger quantities of **3** for biological evaluation. While **3** has impressive CFTR corrector activity in combination with AbbVie potentiator and C1-corrector compounds, it is very lipophilic, which can correlate to diminished drug-like properties.^27,28^ To reduce cLogD, we decided to target heteroaryl modifications of the core cyclopropane motif.^29^

Unfortunately, the Rh_2_(*R*-DOSP)_4_ conditions that worked well for the parent system failed to deliver acceptable yields and sufficient asymmetric induction in cyclopropanations with vinyl heteroarenes of interest. At this stage, we reached out to Prof. Davies for his consultation on this problem, initiating a very fruitful industry-academia partnership stemming from the various precompetitive research projects the teams had already engaged in (*vide supra*). During our first discussion, we were directed to his early study prior to development of the chiral dirhodium catalysts on chiral auxiliary-mediated cyclopropanations^30^ and later confirmed that (*R*)-pantolactone esters provided high asymmetric induction in cyclopropanations with diverse vinylheteroarenes (Figure 4). This approach enabled more rapid assessment of SAR around the cyclopropane C2-substituent but was still limited by the necessity of installing and removing a chiral auxiliary.

**Figure 4:**
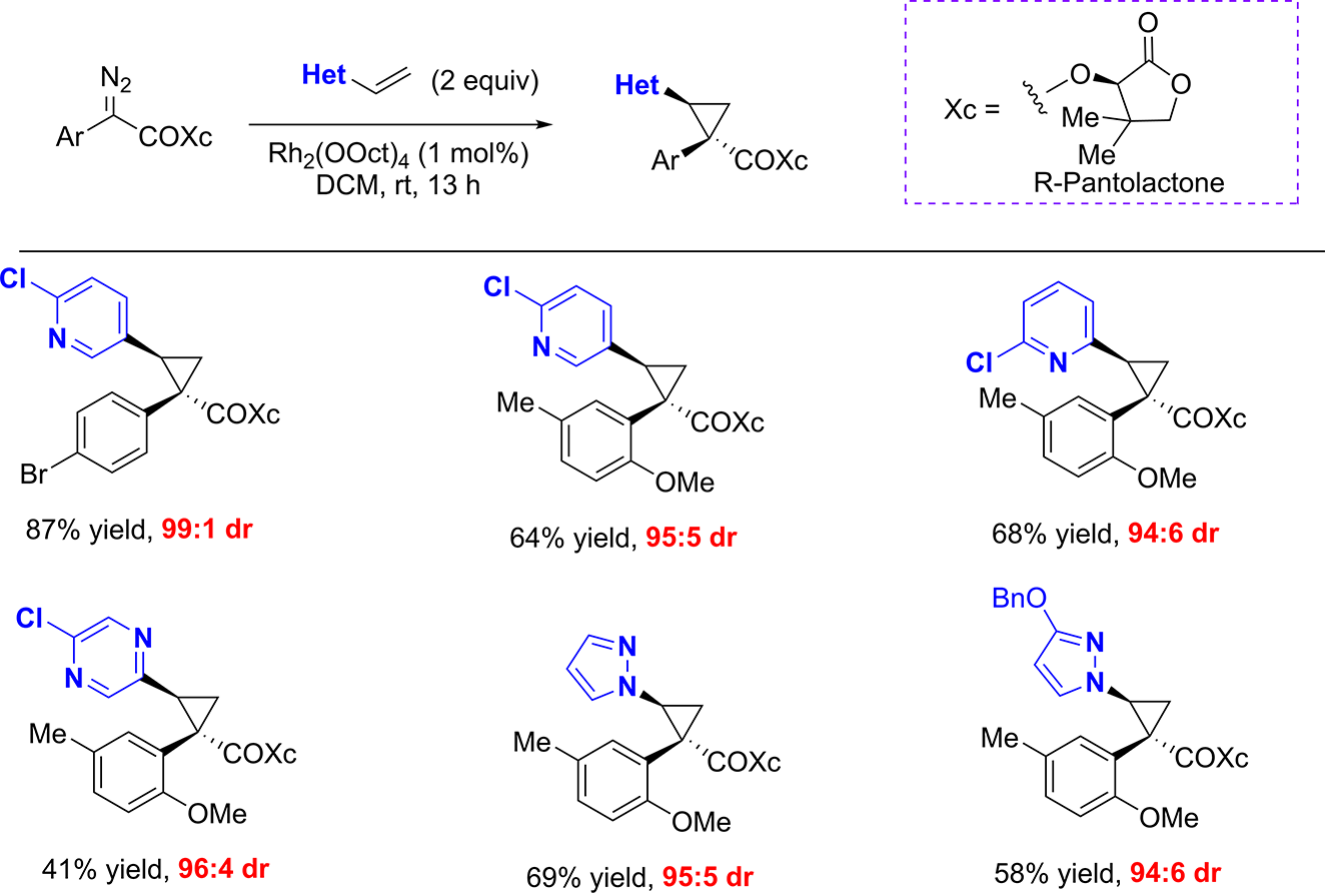
Asymmetric cyclopropanation using *R*-pantolactone as a chiral auxiliary.

Despite the synthetic inefficiencies, several important SAR lessons emerged from these explorations of diarylcyclopropane CFTR correctors (Figure 5). Many heteroaryl derivatives that we synthesized did achieve lower cLogDs than their phenyl counterparts, but the corrector activity trended weaker and was sensitive to substitution and heteroatom positioning within the ring. For example, the unsubstituted pyridine **10** is much less effective as a corrector than phenyl derivative **3** but the activity can be partially restored by introducing halo or alkoxy groups adjacent to the ring nitrogen atom (compare **11** and **12**). Similar trends were observed with other 6-membered heteroaromatic rings, including pyrimidine and pyrazine (**13**–**15**). Overall, the best balance of properties was achieved in the ethoxypyridine analog **12**, later known as ABBV-602.

**Figure 5:**
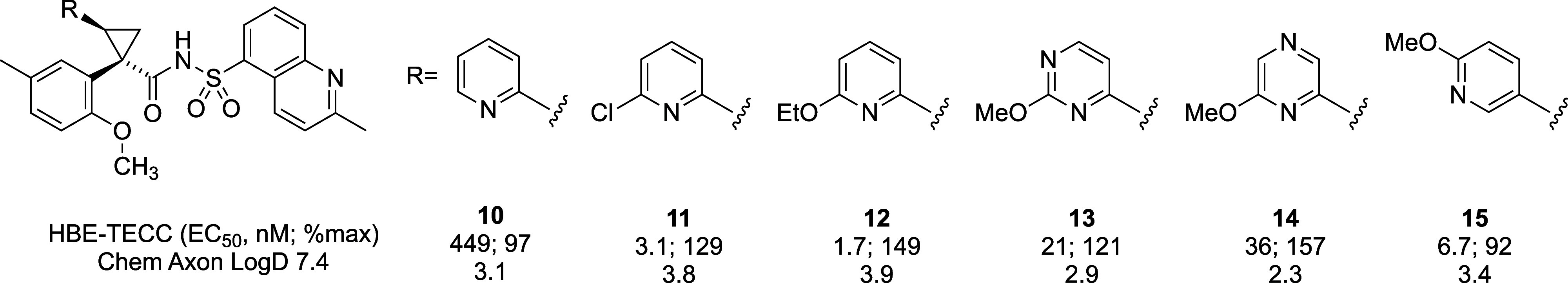
Further refinement of SAR studies identified **12** (ABBV-602) as the lead compound.

As promising compounds progressed through the screening funnel, it became imperative that a practical synthetic method be developed using chiral catalysts instead of a chiral auxiliary. The Davies group has now developed an extensive series of chiral dirhodium catalysts, so we entered into a focused project to identify a suitable chiral catalyst for the desired system. The Davies catalysts have been shown to be routinely capable of high asymmetric induction with a variety of aryldiazoacetates, but ironically the ortho-substituted derivatives required in our system turned out to be problematic substrates. Consequently, the two teams engaged in biweekly collaborative meetings to navigate finding an effective solution to this time-sensitive problem.^31^

A screen revealed the optimal catalyst to be Rh_2_(*S*-TPPTTL)_4_, recently developed by the Davies group for site selective C–H functionalization (Figure 6B).^32^ However, in the initial studies the enantioselectivity was still variable and it was difficult to obtain reproducible results. Extensive troubleshooting of the reaction by both teams resulted in the discovery that the enantioselectivity of this reaction was very sensitive to trace moisture. This was a new phenomenon for these cyclopropanation reactions. The presence of water would have been expected to cause a lowering in the yield due to competing O–H insertion, but typically the level of enantioselectivity in the published cyclopropanation reactions had been very robust. Once the moisture sensitivity had been identified, it was found that consistent asymmetric induction could be achieved by adding large quantities of 4 Å molecular sieves (10 equiv) to the reaction (Figure 6C). Unfortunately, at even modest scales the sheer volume of sieves required for reproducibility was untenable. We then discovered that substituting HFIP (10 equiv) as an additive proved equally effective for retaining asymmetric reproducibility of the reaction and was significantly more practical. Additionally, it was observed that the level of asymmetric induction was curiously enhanced for substrates featuring a chloropyridine substituent. This led to the discovery that routinely high levels of asymmetric induction could be obtained for a broad set of substrates when 3.5 equiv of 2-chloropyridine was included in the reaction (Figure 6D). Mechanistic studies indicated that the 2-halopyridine coordinated to the second rhodium in the dirhodium and this influences the orientation of the chiral ligands in the complex.^31^ Later, for scale-up practicality and safety, 2-fluoropyridine was substituted for 2-chloropyridine, which also allowed a decrease to 1.1 equiv of additive.

**Figure 6:**
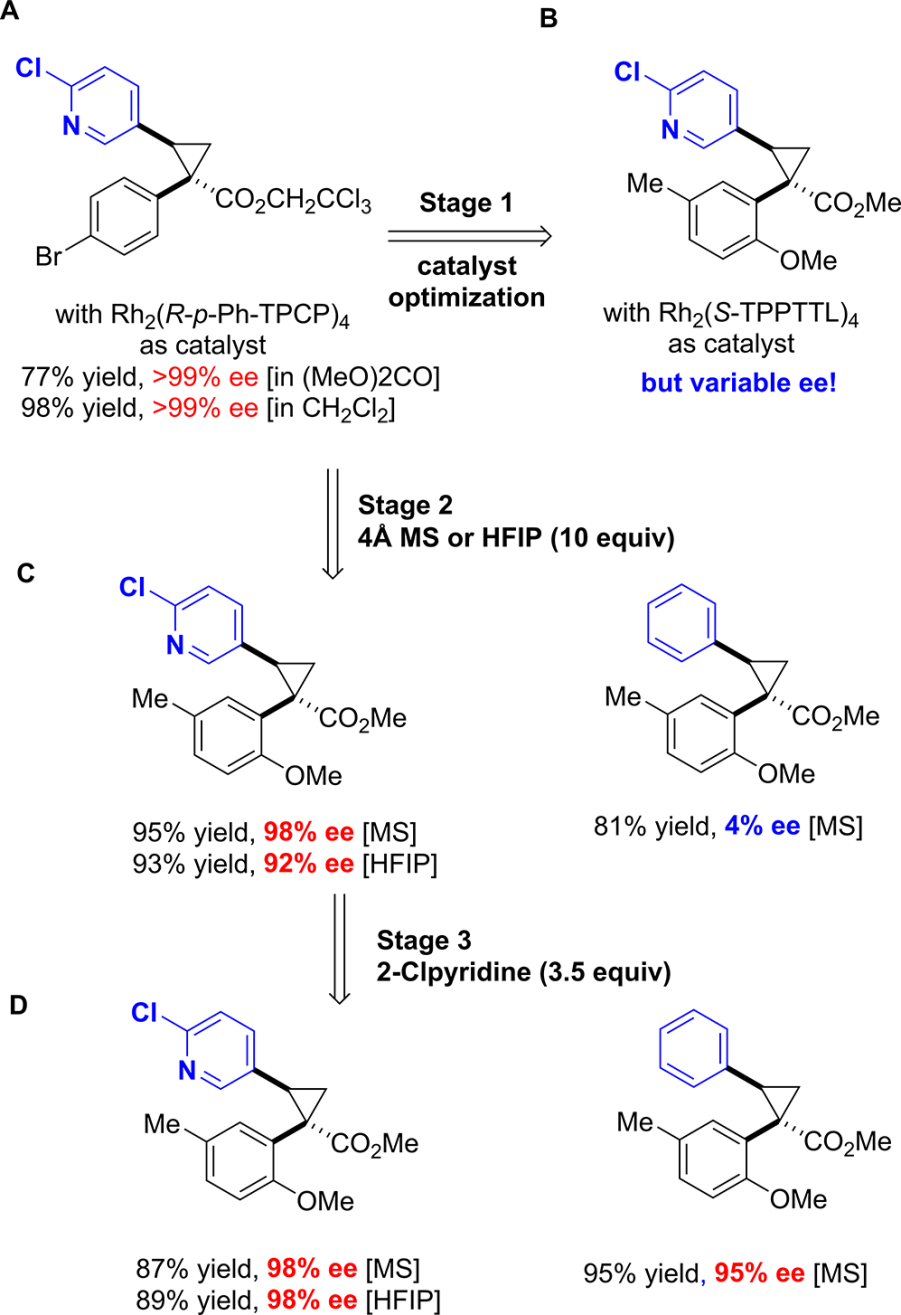
Optimization studies of the enantioselective cyclopropanation of ortho-substituted aryldiazoacetates.^31^ A. Standard cyclopropanation—highly enantioselective cyclopropanation with p-substituted aryldiazoacetate with Rh_2_(*R*-*p*-Ph-TPCP)_4_ as catalyst. B. Catalyst optimization identified Rh_2_(*S*-TPPTTL)_4_ as the optimum catalyst, but the enantioselectivity was variable and irreproducible. C. Use of large amounts of MS or 10 equiv of HFIP resulted in stable enantioselectivity but a large difference was observed between compound having 2-chlorpyridyl functionality present or not. D. Addition of 3.5 equiv of 2-chloropyridine to the reaction causes all the reactions to proceed with high levels of enantioselectivity.

To enable advanced preclinical characterization of ABBV-602, we initiated a 50 g scale-up campaign, utilizing the catalytic asymmetric cyclopropanation developed from our collaboration as the key step (Scheme 1). Starting from the hydrazones **16** (a mixture of E/Z isomers, prepared from commercially available pyruvate), we prepared the diazo intermediate **17** either using Et_3_N or DBU as the base. The crude diazo compound **17** was directly used for asymmetric cyclopropanation with vinylpyridine **18**, requiring only 0.15 mol % Rh_2_(*S*-TPPTTL)_4_ as the catalyst to deliver the chiral trisubstituted cyclopropane **19** (95% ee). Used as crude, ester **19** was hydrolyzed with NaOH to the corresponding acid, which was directly coupled with quinolinyl sulfonamide **21** to give ABBV-602 **12** as an enantiopure white powder following crystallization (52.8 g, 74% overall yield from tosyl hydrazones **16**, >99% ee).

Upon advancement of CFTR C2 corrector candidate ABBV-602, numerous routes were assessed to avoid the use of a diazo intermediate on large scale. However, no alternatives rivaled the efficiency of the asymmetric cyclopropanation. Therefore, flow chemistry was implemented to address safety concerns related to the energetic decomposition of diazoester **17** at relatively low temperatures, minimizing accumulation and improving heat transfer. To facilitate this, mesityl hydrazone **22** was used to accelerate diazo formation at lower reaction temperature. Additionally, a switch from 2-chloropyridine to 2-fluoropyridine as additive in the fed batch asymmetric cyclopropanation reaction was employed allowing for easy removal of this additive via reduced pressure distillation. These changes were implemented to enable synthesis of **12** in 74% yield and 98% ee on 100 g scale in a lab scale flow reactor (Figure 7).^33^ Similar flow conditions were ultimately utilized to provide up to 1.9 kg of **12** in a single fed-batch flow process to enable large scale preparation of ABBV-602.

**Scheme 1:**
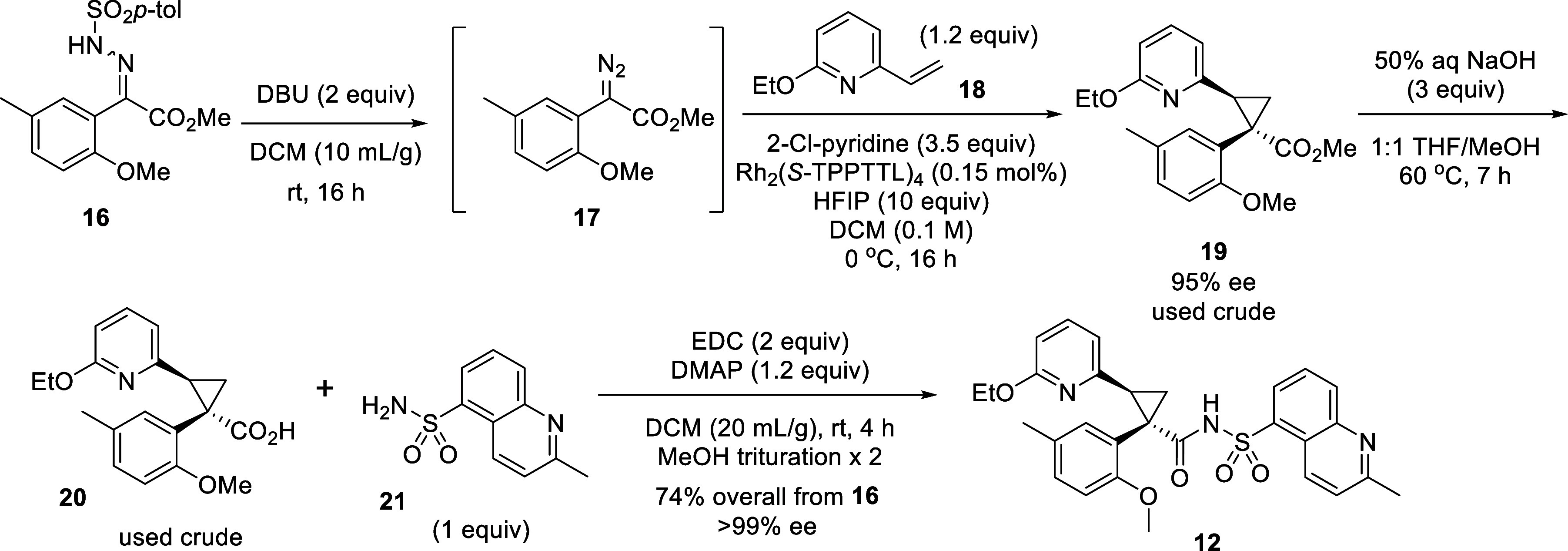
Batch Synthesis of >50 g ABBV-602

**Figure 7:**
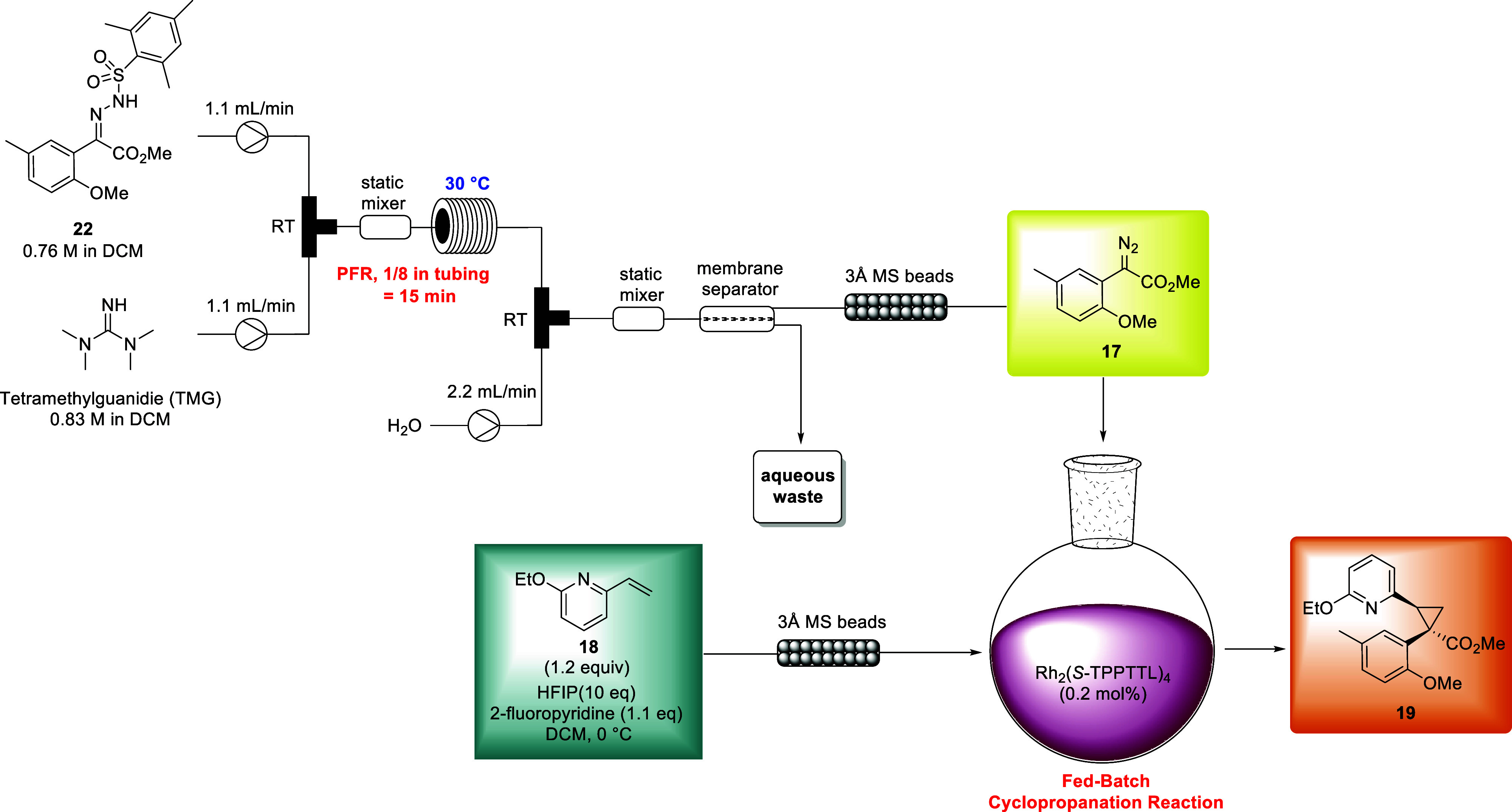
Flow chemistry implemented for asymmetric cyclopropanation.

In conclusion, a long-standing precompetitive collaborative relationship between AbbVie and the Davies group enabled impactful real-time contributions to the discovery of CFTR C2 corrector candidate ABBV-602. Limitations were identified in the existing published methods for asymmetric cyclopropanation, most notably with respect to the tolerance of pharmaceutically relevant nitrogen-containing heterocycles. Catalyst and additive screening unveiled a unique system to overcome these limitations and addressed a specific and pressing need for the AbbVie medicinal chemistry team. This enabled and accelerated unique target synthesis, SAR exploration by iterative compound design, candidate identification, 50 g fit-for-purpose delivery, and ultimately kg-scale synthesis utilizing flow chemistry, which in turn had a profound impact on meeting project timelines and goals.

By fostering collaborative dialogue and research projects in organic synthesis between industrial and academic research groups via platforms such as the Catalysis Innovation Consortium, productive and impactful drug discovery advances are made possible. Furthermore, the chemical challenges faced in these types of collaborations open up new fundamental research opportunities. For example, the discovery that HFIP can protect reactions from traces of water has led to its use to protect C–H functionalization and cyclopropanations from interference by nucleophiles^34,35^ and a recognition that HFIP has a dramatic influence on the conformational mobility of the dirhodium catalysts.^36^ We anticipate an increased occurrence of these projects as historical barriers between industry and academia are addressed in our shared mission to help patients in need of care.

## Abbreviations

CCHF: NSF Center for Selective C–H Functionalization
CIC: Catalysis Innovation Consortium
CF: cystic fibrosis
CFTR: cystic fibrosis transmembrane conductance regulator
SAR: structure activity relationship
SFC: supercritical fluid chromatography
Rh_2_(*R*-DOSP)_4_: tetrakis­[(R)-(+)-N-(p-dodecylphenylsulfonyl)­prolinato]­dirhodium­(II)
HFIP: 1,1,1,3,3,3-hexafluoroisopropanol
MS: 4 Å molecular sieves
HBE-TECC: human bronchial epithelial transepithelial current clamp
cLogD: calculated logD (ChemAxon)
